# Effectiveness of a Load-Imposing Device for Cyclic Stretching of Isolated Human Bronchi: A Validation Study

**DOI:** 10.1371/journal.pone.0127765

**Published:** 2015-05-26

**Authors:** Morgan Le Guen, Emmanuel Naline, Stanislas Grassin-Delyle, Philippe Devillier, Christophe Faisy

**Affiliations:** 1 Research Unit UPRES EA220, University Versailles Saint–Quentin, Hôpital Foch, 40 rue Worth, F-92150, Suresnes, France; 2 Departement of Anesthesiology, Hôpital Foch, 40 rue Worth, F-92150, Suresnes, France; 3 Medical Intensive Care Unit, Hôpital Européen Georges Pompidou, Assistance Publique-Hôpitaux de Paris, University Sorbonne Paris Cité, 20 rue Leblanc, F-75908, Paris, Cedex 15, France; Queen Mary University of London, UNITED KINGDOM

## Abstract

**Background:**

Mechanical ventilation may induce harmful effects in the airways of critically ill patients. Nevertheless, the effects of cyclic stretching caused by repetitive inflation-deflation of the bronchial compartment have not been well characterized in humans. The objective of the present study was to assess the effectiveness of a load-imposing device for the cyclic stretching of human bronchi.

**Methods:**

Intact bronchial segments were removed from 128 thoracic surgery patients. After preparation and equilibration in an organ bath, bronchi were stretched repetitively and cyclically with a motorized transducer. The peak force imposed on the bronchi was set to 80% of each individual maximum contraction in response to acetylcholine and the minimal force corresponded to the initial basal tone before stretching. A 1-min cycle (stretching for 15 sec, relaxing for 15 sec and resting for 30 sec) was applied over a time period ranging from 5 to 60 min. The device's performance level was assessed and the properties of the stretched bronchi were compared with those of paired, non-stretched bronchi.

**Results:**

Despite the intrinsic capacities of the device, the targets of the tension adjustments remained variable for minimal tension (156–178%) while the peak force set point was unchanged (87–115%). In the stretched bronchi, a time-dependent rise in basal tone (*P* <.05 vs. non-stretched) was apparent after as little as 5 min of cyclic stretching. The stretch-induced rise in basal tone continued to increase (*P* <.01) after the stretching had ended. Only 60 min of cyclic stretching was associated with a significant (*P* <.05) increase in responsiveness to acetylcholine, relative to non-stretched bronchi.

**Conclusions:**

Low-frequency, low-force, cyclic loading of human bronchi is associated with elevated basal tone and acetylcholine responsiveness. The present experimental model is likely to be a useful tool for future investigations of the bronchial response to repetitive stress during mechanical ventilation.

## Introduction

The human bronchi are constituted by a complex network of cells and tissue structures: rings of cartilage, fibrous tissue, elastic fibers, muscle fibers, blood vessels, epithelial and glandular cells, nerves, and inflammatory cells. The lung can be viewed as a tissue in which the cells must adapt to deformation of the scaffolding to which they adhere. Like other lung tissues, the bronchi undergo cycles of stretching and shortening during inspiration and expiration, respectively. Both spontaneous breathing and mechanical ventilation impose a cyclic tension on the trachea/bronchial tree. In positive-pressure ventilation (the most usual mode of mechanical ventilation), positive pressure is directly transmitted to the apical side of the bronchial cells. It is known that the response to focal, homogeneous application of pressure to the apical side of airway smooth muscle (ASM) cells differs from that evoked by the application of longitudinal force, since specific signaling pathways are triggered in each case [1,2]. In general, isolated cell models have been used to study the impact of cyclic stretching on the airways, whereas integrated models have rarely been studied (with the exception of tracheal segments) [3]. However, the generalization of mechanical ventilation in surgical or intensive care settings has prompted characterization of the clinical impact of inflation-deflation on the alveolar compartment [4,5] but not on the proximal airways.

The effects of mechanical ventilation have been investigated in vitro by applying cyclic stretching to alveolar epithelial cells and alveolar macrophages [6,7]. Moreover, most studies of the impact of stretching on ASM have been based on isolated tissue strips taken from animals; the corresponding results indicate that ASM's responsiveness is largely attenuated by dynamic stretching. The intact airway is more geometrically and structurally complex than isolated airway smooth muscle strips, which may decrease the latter's relevance. We have previously reported that a single, manually controlled, static loading of isolated human bronchi for 5 consecutive min (at a force corresponding to an inflation pressure of over 30 cm H_2_O) profoundly altered the crosstalk between airway cells by eliciting contractile and pro-inflammatory mediators and inducing a myogenic response [8]. This single, extraphysiological application of tension to human bronchi provoked a two-step increase in basal tone (involving pro-inflammatory mediators in the early phase and the Rho-A kinase pathway and wingless-type MMTV integration site family protein (WNT) gene regulation in the late phase). In mice, mechanical ventilation increases the volume of the upper airways by a factor of 2.5 (relative to spontaneous breathing) and triggers the release of pro-inflammatory cytokines by the trachea [9]. However, our single-loading model is not suitable for investigating the effect of prolonged periods of repetitive loading on whole human bronchi. Furthermore, the inflation pressure is not necessarily correlated with airway responsiveness (i.e. the bronchi's primary function). Hence, the main objective of the present study was to assess the effects of a load that was proportional to each bronchus' intrinsic contractile properties. Load was imposed by a new cyclic stretching device developed in house for this purpose. The study's primary outcome measure was the device's technical efficiency and the secondary outcome measure was the bronchial segment's response to non-physiological cyclic stretching.

## Materials and Methods

### Patients

The study's protocol and consent procedure were approved by the local investigational review board (*Comité de Protection des Personnes Ile de France VIII*, Boulogne-Billancourt, France). At least one day before scheduled lung cancer surgery, patients gave their written informed consent to participation (which was noted in the individual's medical files).

### Human bronchus preparation

All bronchoactive medications were withdrawn the evening before surgery. A bronchial tissue sample was obtained from each of 129 patients. Bronchial segments judged to be macroscopically free of cartilaginous tissue were excised as far as possible from the malignant lesion (the absence of tumor infiltration was retrospectively established in all cases) and were immediately immersed in and washed thoroughly with oxygenated Krebs-Henseleit solution (NaCl 119 mM, KCl 4.7 mM, CaCl_2_ 2.5 mM, KH_2_PO_4_ 1.2 mM, NaHCO_3_ 29 mM, and glucose 11.7 mM) to remove any intraoperative drug residues. Bronchial rings of similar length (5 mm) and inner diameter (1–2 mm) were prepared by removing adhering lung parenchyma and connective tissue and were then washed in oxygenated Krebs-Henseleit solution.

### Structure and configuration of the experimental device

**Fig 1:**
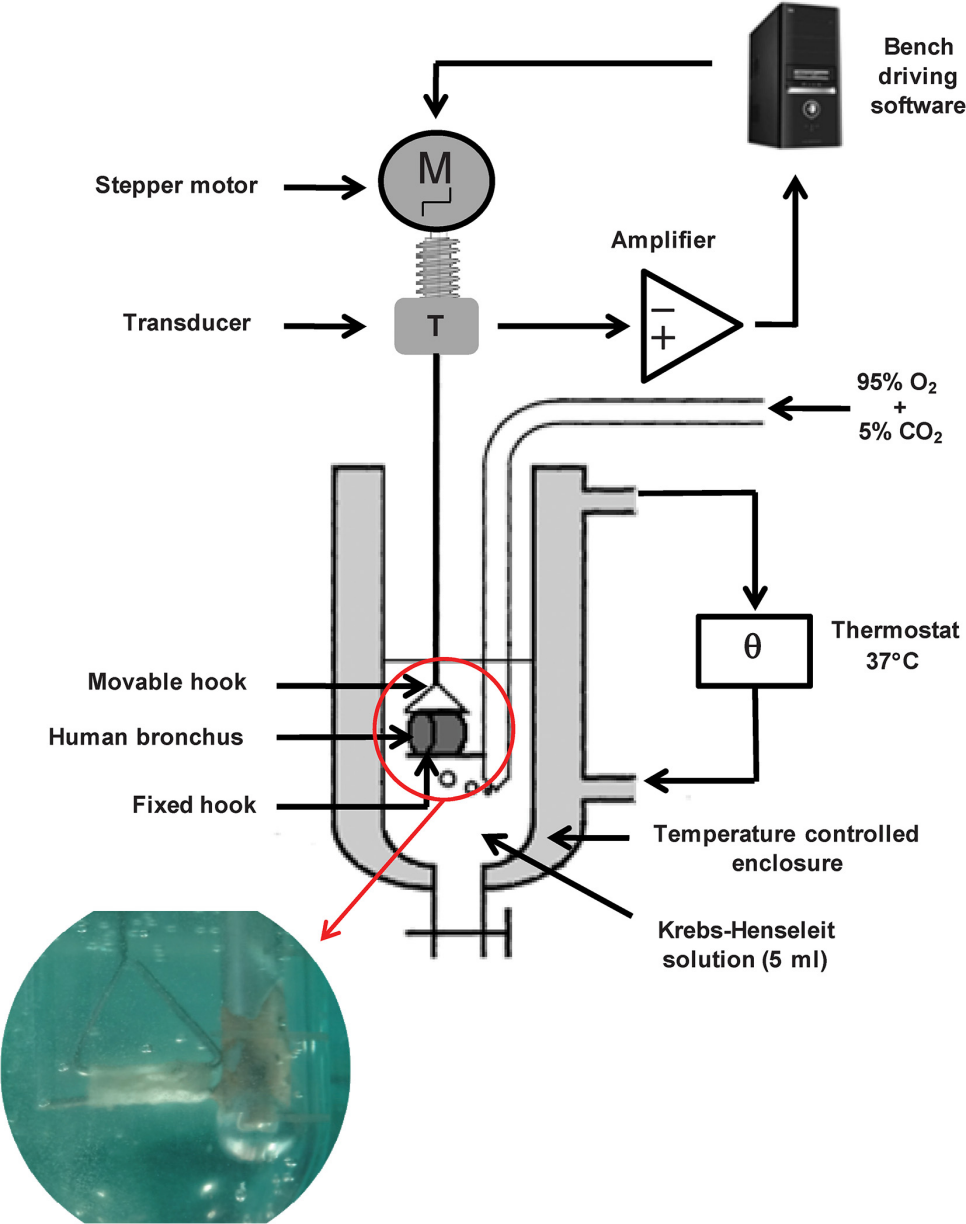
The experimental device for the cyclic stretching of isolated human bronchi.

The bronchial rings were hung horizontally on a stainless steel hooks that passed through the lumen. The rings were then placed in a 37°C organ bath containing 5 ml of Krebs-Henseleit solution gassed with 95% O_2_/5% CO_2_ (Fig 1). One hook was fixed to the core holder at the bottom of the organ bath and another (triangle-shaped) hook was fastened to an isometric force transducer (it1, Emka Technologies, Paris, France) mounted on a motorized Vernier micropositioner (Emka Technologies). This positioner was connected to a tissue tension controller (Emka Technologies) that enabled precise, automatic adjustments of the tension. The transducer was connected to an amplifier and the tension data were acquired, processed and analyzed using IOX (version 2.4.2) and Datanalyst (version 2.1.0) software packages (EMKA Technologies).

**Fig 2:**
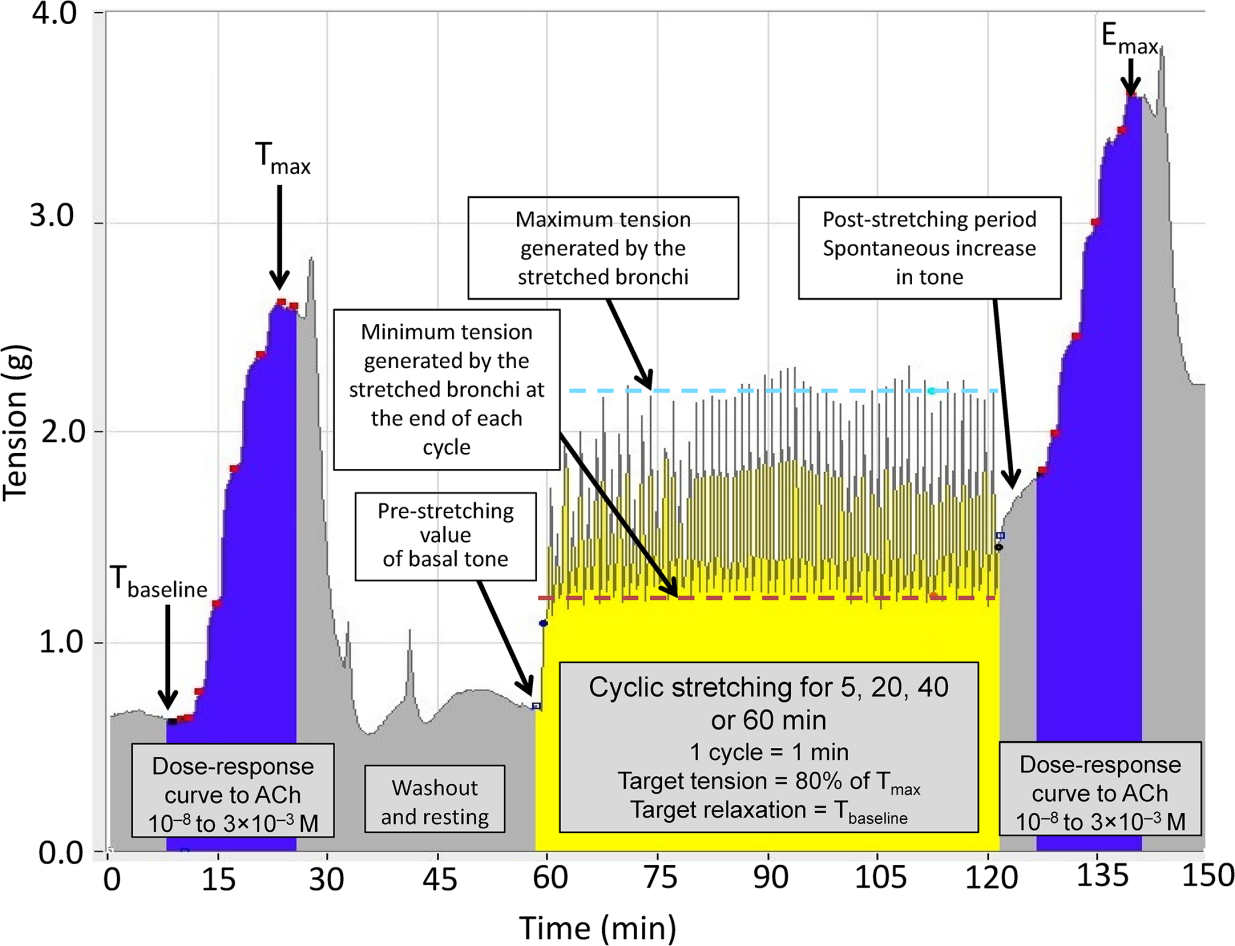
The experimental protocol for the cyclic stretching of human bronchial rings in a 37°C organ bath containing 5 ml of Krebs-Henseleit solution gassed with 95% O_2_/5% CO_2_. The illustration is based on data captured from an actual experiment. T_**max**_: maximum bronchial contraction in response to 3 × 10^–3^ M acetylcholine (ACh) before cyclic stretching; T_**baseline**_, resting basal tone. T_**baseline**_ and 80% of T_**max**_ were used to define the settings for cyclic stretching. The expected tension variation generated by the device (80% T_**max—**_T_**baseline**_) was compared to the observed values generated by the bronchial rings (maximum tension—minimum tension) in response to the forces imposed by the device (Table 2).The initial test of bronchial reactivity with 3.10^–3^ M acetylcholine was not represented.

The cyclic stretching device was developed by modifying a conventional, manual organ bath device. Automatically controlled stretching was enabled by connecting a tissue tension controller to the motorized micropositioner. This allows continuous measurement of the isometric contraction developed by the tissue and the application of automated, standardized variations in tissue tension (Fig 1) with an acceptable variability of ± 10%. The automatic tension controller's main characteristics are indicated in Table 1. The stretching cycle lasted for 1 min and comprised three phases. In the first (15-sec) phase, the tension applied to the tissue is increased to 80% of the maximum tension (T_max_) developed by the bronchial ring (see below). This level of tension corresponded approximatively to twice or three times the basal tone, i.e., an airway inflation pressure of 30 cm H_2_O [8], limiting the risk of bronchial damage. The increase in tension was divided into two subphases: a steep increase to just below the target value (lasting 10 sec), followed by a slower increase to the target value over the last 5 sec. These two phases mimic the increase in insufflated volume during volume-assisted ventilation in clinical practice. The stretching period was followed by a 15-sec phase during which the tension returned to the baseline tension (T_baseline_). Lastly, no change in tension was applied to the tissue for 30 sec (Fig 2). The tension at the end of each of the three phases was automatically recorded by the system. In the stretched group of rings, cyclic stretching consisted in the repetition of the 1-min cycle for various time periods (5, 20, 40 and 60 min). A dedicated algorithm was built to automate the stretching sequences described above. Experiments on non-stretched controls (i.e. rings at baseline tone) and stretched groups of rings were run in parallel.

**Table 1:** Technical characteristics of the automated cyclic stretching controller.

	Range of values	Extreme values	Precision
Basal tone, g	1–3	0–12	0.001
Maximum developed tension, g	2–7	0–12	0.001
Speed of linear displacement, mm/sec	1–1.25	0.75–1.25	0.10
Measurement frequency (Hz)	10	0.1 to 60	not applicable

The "extreme values" and "precision" correspond to data provided by the device's manufacturer. The "range of values" corresponds to the experimental range chosen by the investigators with a view to optimizing feasibility and facilitating data collection.

### Experimental procedure

Bronchi were suspended on the hooks with a preload of 1.5 g (in view of their small inner diameter) [10] and were equilibrated for at least 60 min in Krebs-Henseleit solution, which was changed every 15 min. At the end of the equilibration period, the reactivity of the bronchial rings was then tested with 3 × 10^–3^ M acetylcholine (ACh), the concentration that caused maximum contraction (i.e. maximum developed tension (T_max_)). When the contraction reached a plateau, the tissues were washed every 10 min until the basal tone spontaneously returned to T_baseline_ (Fig 2). During this second equilibration period, the tissue tension-controller set-point tensions were adjusted according to the rings' individual T_baseline_ and T_max_ values. Next, a first cumulative ACh dose-response curve (10^–8^ to 3 × 10^–3^ M) was established, in order to characterize the bronchial ring's responsiveness profile (Fig 2). After a plateau was reached with the maximum concentration of ACh, the tissues were again washed every 10 min until the tension returned to T_baseline_. Bronchial ring from a given patient were randomly allocated to the stretched or non-stretched experimental group. When the period of stretching had ended, the ring was left to equilibrate in Krebs-Henseleit solution for 15 min with no imposed tension. A second cumulative ACh dose-response curve (10^–8^ to 3 × 10^–3^ M) was established (Fig 2). Lastly, the bronchial rings were patted dry and weighed.

### Validation of the experimental model

In a first series of 40 human bronchi, the optimal duration of cyclic stretching was assessed by analyzing the results for four different stretching durations (5 min, 20 min, 40 min and 60 min) on corresponding sets of 10 bronchi. The optimal stretching time was established to be 60 min, since the measured tension was found to remain stable after 20–40 min of stretching. Given that the T_baseline_ and T_max_ values of human bronchial rings and their predictors were not known, we applied Maxwell’s significance rule [11]; this states that a series of 50 experiments is needed to achieve acceptable statistical power. Therefore, a second series of experiments was performed on human bronchi (*n* = 79). To rule out the possible stretch-induced secretion of contractile mediators into the organ bath [8], an additional wash was performed during the post-stretching period (10 min after the end of the stretching cycles) on 10 additional paired human bronchi.

### Data analysis and statistics

Data are presented as the mean ± SEM (*n)*, where n is the number of subjects, or as the standardized effect size *d* [95% confidence interval]. Basal tone and contractile responses are expressed as tension (g). To validate the device's performance, the set-point values [80% T_max_, T_baseline_], the expected tension variation generated by the device [80% T_max—_T_baseline_] and the observed values generated by the bronchial rings in response to the forces imposed by the device were compared (Fig 2). ACh's efficacy (T_max_/E_max_, g) corresponds to the maximum contraction induced by 3 × 10^–3^ M ACh. ACh's potency (-log EC_50_) was defined as the negative log of the ACh concentration that induced 50% of the maximum effect. ΔT_max_/ΔE_max_ (g) represented the difference between T_max_/E_max_ obtained with 3 × 10^–3^ M ACh and the basal tone (T_baseline_/T_post-stretch_) recorded just before establishment of the dose-response curve. The effect of cyclic stretching on bronchial basal tone was assessed immediately after the cyclic stretching had ended and also 10 min after the stretching ended. The effect of cyclic stretching on the human bronchial rings' responsiveness to ACh was assessed by measuring efficacy (expressed as ΔT_max_/ΔE_max_) and potency (as determined by the concentration-response curves) before and after the stretching period (Fig 2). The results were analyzed using Student's t test for paired data or a one-way repeated-measures analysis of variance, as appropriate. All statistical analyses were performed with StatView software (version 5.0, SAS Institute Inc. Cary, NC). The threshold for statistical significance was set to *P* <.05. The standardized effect size *d* for the difference between means was calculated to determine whether the observed effect of stretch or organ bath washout was small (|*d*| ≥.20), medium (|*d*| ≥.50) or large (|*d*| ≥.80), according to Cohen’s conventions [12]. The 95% confidence interval (CI) for *d* was calculated in order to determine the level of uncertainty associated with the estimate of the effect size.

## Results

### Sample

In the first series of human bronchi (*n* = 40), one experiment was not completed (due to the rupture of a bronchial ring in the 5-min stretched group) and was therefore excluded from our analysis. All the data from the validation series (*n* = 79) and from the series with an additional post-stretching wash (*n* = 10) were analyzed. Overall, data obtained for bronchial rings from 128 patients (82 men and 46 women, all ex-smokers; mean ± SEM age: 58±10) were included in the analysis. The mean patted-dry weight of the stretched bronchial rings was similar to that of the paired, non-stretched controls (26±0.6 vs. 27±0.7 mg, respectively; *n* = 128, *P* = 0.29).

### Technical performance of the automated, cyclic stretching and validation of the model

The mean T_baseline_ and T_max_ values were respectively 1.03±0.06 g and 2.98±0.38 g. The tension generated by the bronchi in response to the variations imposed by the device was 2.59±0.11 g for the maximum tension at the end of the first phase of the automated increase in tension and 1.90±0.07 g for the minimal tension at the end of the second phase of automated decrease in tension. The tensions measured during the initial stretching cycles were highly variable but then stabilized (to less than 10% difference between two successive measurements) after 3.5±0.5, 7.5±0.5, 5.5±0.5 and 7.0±0.5 cycles for total cyclic stretching times of 5, 20, 40 and 60 min, respectively. The main finding was the immediate increase in the minimal tension at the end of the stretching cycles, with a value well above T_baseline_ or the pre-stretch baseline tone (Fig 2). A large difference between the minimal tension reached at the end of each stretching cycle and the minimal setting value (T_baseline_) was observed, irrespective of the total cyclic stretching time (Table 2). The mean differences between measured tensions and preset values are shown in Table 2. In general, the minimum tensions generated by the stretched bronchi were higher than the pre-stretch values observed in the very first cycles. However, after 40 min of stretching, the maximum tension plateaued at values close to the maximum preset values (T_max_); accordingly, the measured tension variation (i.e. the difference between measured minimum and maximum tensions) was about half the device's preset values (Table 2).

**Table 2:** Performance of the automated stretching system on human bronchi.

	Overall duration of cyclic stretching	*n*	Differences between measured and preset values	
			g	%
T_baseline_				
	5 min	9	0.47±0.09**	178±28
First series	20 min	10	0.58±0.09**	164±12
	40 min	10	0.52±0.09**	161±12
	60 min	10	0.37±0.09**	156±21
*Second series (validation)*	60 min	79	0.42±0.05**	160±10
T_max_				
	5 min	9	-0.53±0.27*	87±10
First series	20 min	10	-0.19±0.29	96±11
	40 min	10	0.00±0.21	109±8
	60 min	10	0.23±0.26	115±10
*Second series (validation)*	60 min	79	0.06 ±0.06	106±3
Tension variation (T_max—_T_baseline_)				
	5 min	9	0.84±0.12**	43±9
First series	20 min	10	0.35±0.15*	52±9
	40 min	10	0.15±0.22	58±12
	60 min	10	0.13±0.21	88±21
*Second series (validation)*	60 min	79	0.14±0.13	55±5

Data are shown as the mean ± SEM. The imposed tension was measured every 0.1 sec with a dedicated software package (IOX, version 2.4.2, EMKA Technologies, Paris, France). The magnitude of the stretch generated by the system corresponded to the difference between 80% T_max_ and T_baseline_ (Fig 2). The expected tension variation generated by the device (80% T_max—_T_baseline_) was compared to the observed values generated by the bronchial rings (maximum tension—minimum tension) in response to the forces imposed by the device (Fig 2).

*P <.01

**P <.001 when comparing measured and preset values.

### The effect of cyclic stretching on bronchial basal tone

**Fig 3:**
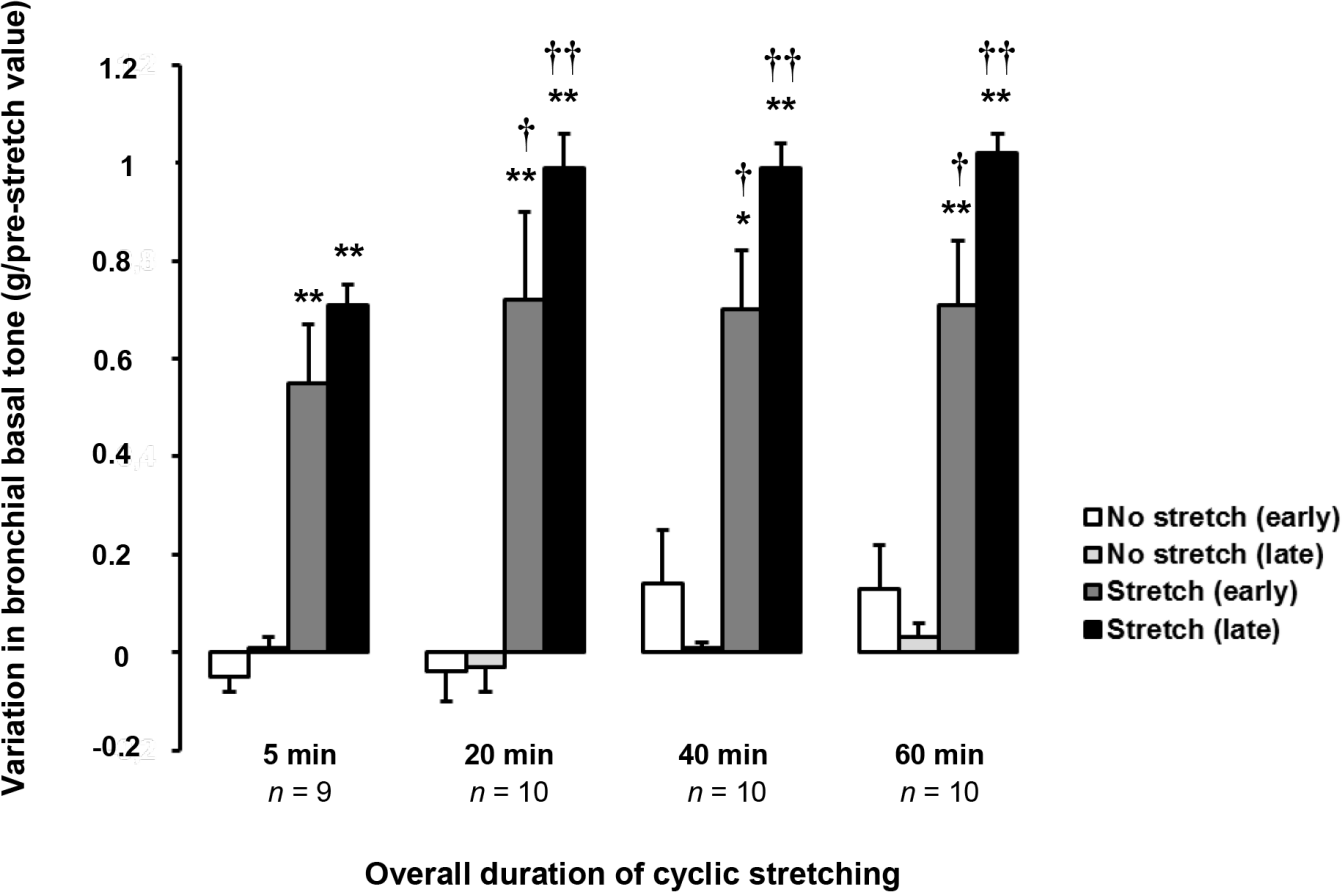
Effect of the duration of cyclic stretching on airway basal tone. The pre-stretching basal tone was recorded immediately before the onset of cyclic stretching (Fig 2). The variation in basal tone corresponds to the deviation from the pre-stretching value. The early stretching-induced effect corresponds to the change in bronchial basal tone at the start of the cyclic stretching (Fig 2). The late post-stretching effect corresponds to the spontaneous increase in basal tone after the cessation of cyclic stretching (until basal tone reaches a plateau after 10 min (Fig 2). Data are quoted as the mean ± SEM. *P <.05, **P <.01 vs. paired non-stretched control; ^†^ *P* <.05, ^††^ *P* <.01 vs. 5 min of cyclic stretching. See Table 3 for details.

After the early increase in basal tone (0.82±0.06 g, *n* = 79), cyclic stretching triggered a second (late) post-stretching increase in basal tone (0.28±0.02 g, *n* = 79) immediately after the end of the stretching period. The basal tone reached a plateau in about 10 min (Fig 2 and Fig 3 and Table 3). The post-stretching increase in basal tone varied according to the total cyclic stretching time and reached its greatest value after 20 min of stretching (Table 3, Fig 3). In control bronchi, basal tone did not change significantly over the same period of time.

**Table 3:** Effect of cyclic stretching on bronchial basal tone.

	Overall duration of cyclic stretching	*n*	Variation in basal tone (g)		Effect size
			Control	Stretched	|*d*| [95%CI]
Early stretch-induced effect					
	5 min	9	-0.05±0.03	0.55±0.12**	6.86 [4.2–8.9]
*First series*	20 min	10	-0.04±0.06	0.72±0.18**	5.66 [3.5–7.3]
	40 min	10	0.14±0.11	0.70±0.12*	4.86 [2.9–6.3]
	60 min	10	0.13±0.09	0.71±0.13**	5.19 [3.2–6.7]
*Second series (validation)*	60 min	79	0.02±0.02	0.82±0.06***	17.9 [15.8–19.8]
Late post-stretching effect					
	5 min	9	0.01±0.02	0.16±0.04**	4.74 [2.8–6.3]
*First series*	20 min	10	-0.03±0.05	0.27±0.07**	4.93 [3.0–6.4]
	40 min	10	0.01±0.01	0.29±0.05**	7.77 [5.0–9.9]
	60 min	10	0.03±0.03	0.31±0.04**	7.92 [5.1–10.1]
*Second series (validation)*	60 min	79	-0.01±0.01	0.28±0.02***	18.3 [16.2–20.3]

Data are quoted as the mean ± SEM, the standardized effect size (*d*) and the latter's 95% confidence interval (CI) for the difference between means. The early stretch-induced effect corresponds to the change in bronchial basal tone when stretching is initiated (Fig 2). The late post-stretching effect corresponds to the spontaneous increase in basal tone after the cessation of cyclic stretching (until a plateau was reached 10 min later) (Fig 2).

*P <.05

**P <.01

***P <.001 stretched vs. paired non-stretched controls.

### The effect of cyclic stretching on bronchial responsiveness

When compared with paired controls, cyclic stretching did not influence ACh's efficacy but did increase its potency in the first series of experiments (Table 4). Only 60 min of cyclic stretching in the second series of experiments (on a larger number of bronchial preparations) was associated with a statistically significant increase in ACh's efficacy and potency.

**Table 4:** Effect of cyclic stretching on bronchial responsiveness.

	Overall duration of cyclic stretching	*n*	Difference between ACh pre- and post-stretching dose-response curves		Cyclic stretching effect	Effect size
			Controls	Cyclic stretching		|*d*| [95% CI]
*ΔEmax*, *g*						
	5 min	9	-0.27±0.09*	-0.12±0.21	0.15±0.19	0.93 [-0.1–1.8]
*First series*	20 min	10	-0.01±0.14	0.01±0.13	0.02±0.15	0.15 [-0.7–1.0]
	40 min	10	-0.06±0.14	0.06±0.15	0.12±0.25	0.83 [-0.1–1.7]
	60 min	10	-0.16±0.15	-0.02±0.15	0.14±0.22	0.93 [0–1.8]
*Second series (validation)*	60 min	79	0.04±0.04	0.17±0.07*	0.14±0.07^†^	2.28 [1.9–2.7]
*-log EC*_*50*_, *log unit*						
	5 min	9	-0.18±0.07	0.15±0.09	0.33±0.07^††^	4.09 [2.3–5.5]
*First series*	20 min	10	0.07±0.13	0.43±0.13	0.36±0.06^†††^	2.77 [1.4–3.8]
	40 min	10	0.02±0.14	0.59±0.17**	0.58±0.14^††^	3.66 [2.1–4.9]
	60 min	10	-0.07±0.06	0.62±0.14*	0.61±0.12^†††^	6.41 [4.0–8.2]
*Second series (validation)*	60 min	79	0.01±0.03	0.47±0.05***	0.45±0.04^†††^	11.1 [9.8–12.4]

Data are quoted as the mean ± SEM, the standardized effect size (*d*) and the latter's 95% confidence interval (CI) for the difference between means. ACh, acetylcholine; 2ΔEmax (efficacy): the difference between the maximum bronchial contraction obtained with 3 × 10^–3^ M ACh and the basal tone recorded just before the dose-response curve was established (Fig 2);-log EC_50_ (potency), negative log of the ACh concentration producing 50% of the maximum effect.

*P <.05

**P <.01

***P <.001 pre-stretch vs. post-stretch

^†^ *P* <.05

^††^ *P* <.01

^†††^ *P* <.001 stretched segments vs. paired non-stretched controls.

### The effect of an additional organ bath wash after 60 min of cyclic stretching on bronchial basal tone and responsiveness

An organ bath wash performed after 60 min of cyclic stretching did not significantly modify the post-stretching increase in basal tone or responsiveness to ACh in 10 human bronchi (Table 5).

**Table 5:** Effect of organ bath washout after 60 min of cyclic stretching on bronchial basal tone and responsiveness.

Post-stretching effect	No washout	Washout	Effect size
	*n* = 10	*n* = 10	|*d*| [95% CI]
Basal tone, g			
*Before washout*	0.28±0.06	0.34±0.11	–
*Variation induced by washout*	0.03±0.05	0.06±0.04	0.66 (-0.26–1.53)
*ΔEmax*, g	0.17±0.06	0.17±0.06	0 (-0.88–0.88)
-log EC_50_, log unit	4.88±0.54	4.95±0.53	0.13 (-0.75–1)

To eliminate the possible stretch-induced secretion of contractile mediators into the organ bath, a washout was performed (complete renewal of the Krebs-Henseleit solution) after 60 min of cyclic stretching. Data are quoted as the mean ± SEM, the standardized effect size (*d*) and latter's 95% confidence interval (CI) for the difference between means. ACh, acetylcholine; ΔEmax (efficacy), difference between the maximum contraction obtained with 3 × 10^–3^ M ACh and the basal tone recorded just before the second dose-response curve was established (Fig 2);-log EC_50_ (potency), negative log of the ACh concentration producing 50% of the maximum effect (second dose-response curve) (Fig 2). Compared with paired no washout controls, washout did not change the post-stretching basal tone or ACh's efficacy and potency.

## Discussion

The present study shows that cyclic stretching (1 cycle/min, and a maximum tension corresponding to a submaximum bronchial contraction in response to ACh) triggered an increase in basal tone and responsiveness to ACh in a time-dependent manner. The immediate stretch-induced increase in basal tone prevented the bronchial ring from returning to its baseline tension at the end of each stretching cycle. However, the maximum tension generated by the bronchial ring in response to the stretch progressively reached the preset maximum tension imposed by the device and then stabilized after 40 min of cyclic stretching. Axial stretch provokes a decrease in airway smooth muscle force following length perturbation, as a result of cross-bridge detachment and/or reorganization of the cytoskeleton [13,14]. It has also been shown that cyclic radial stretch, corresponding to a physiological airway pressure of 5–30 cm H_2_O, provoked a transient reduction in responsiveness of human isolated bronchi [15]. Supplementing these findings with a large experimental sample, our results indicate that loading of human bronchi with a force orthogonal to their axis induces an increase in basal tone and responsiveness. Such differences may be explained, at least in part, by the non-circumferential (or spiral) organization of ASM in small and medium-sized human bronchi. Therefore, the direction of the forces imposed on bronchi must be accounted for interpreting the impact of cyclic stretching.

The early increase in basal tone after 5 min of stretching is reminiscent of the maintenance of passive stiffness following ASM shortening [16]. Our model highlights the role of airway basal tone as the key effector in the bronchial response to repetitive, imposed, cyclic tension. The observed stretch-induced increase in basal tone suggests that the human bronchi had been sensitized. In healthy subjects, ASM tone is regulated on several different levels: activation of the myosin light chain kinase (MLCK) with enhancement of the phosphorylation, inhibition of the myosin light chain phosphatase (MLCP), which thus decreases dephosphorylation [17–19], and Ca^2+^ sensitization that increases the ASM contraction triggered by a given changes in the intracellular Ca^2+^ concentration [20,21]. Hence, Ca^2+^ sensitization alone cannot explain our observation of a two-phase (early/late) increase in basal tone. We hypothesize that ASM sensitization is probably involved in the early increase in airway tone, as suggested by the concomitant increase in ACh's potency. In fact, one general view is that MLCK is activated in the initial phase of ASM contraction, whereas the sustained phase is maintained by inhibition of MLCP [19]http://ajpcell.physiology.org/content/295/2/C358-ref-33. A phasic mouse bladder muscle has been used to evidence the time course of the constrictive response to carbachol (involving Ca^2+^ sensitization and MLCK activation), with a maximum response as early as 1 min and maintenance of a sustained contractile phase by MLCP inhibition [22]. These findings can be transposed to our human bronchial model of cyclic stretching, in which basal tone increased quickly (in the first 5 min) and then stabilized over the next 20 min of stretching.

When cyclic stretching ceased and the bronchi were no longer subjected to imposed tension, we observed a spontaneous, steady increase in the bronchial tone until a plateau was reached (in about 10 min). We previously found a similar, immediate rise in basal tone after the tension ended when studying human bronchi subjected to a single, 5-min, static loading with a force of 2.5 × basal tone. This increase in basal tone was triggered by the increased epithelial production of leukotrienes following the activation of Ca^2+^-independent inducible nitric oxide synthase, and induced a myogenic response that was dependent on the Rho-kinase and WNT signaling pathways [8]. In the present model of prolonged, cyclic stretching, it remains to be established whether contractile mediators are involved in the post-stretching increase in basal tone. The organ bath wash at the end of the stretch period did not alter the post-stretching increase in basal tone or the responsiveness to ACh. Therefore, the release of endogenous mediators into the bath is unlikely to be involved in this phenomenon. Further investigations of the mechanisms involved in this model are thus required.

Given that the tension imposed on the human bronchi corresponded to 80% of the maximum response induced by ACh, the present model of cyclic loading differs from our previous single-loading model in which the extraphysiological tension corresponded to an airway inflation pressure >30 cm H_2_O [8]. The considerable mechanical plasticity of ASM enables optimal force generation over a broad range of length changes (referred to as "length adaptation") [23]. Furthermore, length oscillations produce a reversible reduction in the amount of myosin filaments; this suggests that rapid myosin assembly and disassembly of the remaining filaments optimizes the developed strength (as observed in the post-stretching period) [24]. The tension imposed on airway cells probably triggers a cascade of signaling events mediated by the macromolecular protein complexes bound to the transmembrane integrins that transduce the external forces from the extracellular matrix to the actin cytoskeleton; this transduction results in gene activation and the production of contractile mediators [25,26]. Cyclic stretching can also activate mechanotransduction in an amplitude- and frequency-dependent manner; this may explain (at least in part) the various observations and interpretations in the literature [27].

In the present experimental model, the bronchial response to cyclic stretching was remarkable because the maximum tension developed by the bronchi exceeded the T_max_ in response to ACh after 20 to 40 min. Cyclic stretching for more than 60 min significantly enhanced ACh's potency but not its efficacy, suggesting that the stretch-induced effect is mainly triggered by the sensitization of the contractile filaments and not by the up-regulation of muscarinic receptors (which is unlikely over such a short period of time). Our results support the hypothesis whereby as many whole contractile units are recruited as possible (as previously described for bovine airway segments subjected to a single 3-min static loading) [28]. After priming the contractile apparatus by pretensioning, the stretch-induced stimulation of the ASM cytoskeleton causes rearrangements of the contractile apparatus, which in turn lead to generation of a force above the pre-stretch level [29]. The ASM's adaptability to elevated muscle tone (by generating more total force) has been already described in experiments on ovine tracheas [30]. The basal tone was increased by the addition of exogenous ACh, and the total force generated in response to the release of endogenous ACh by electric field stimulation (EFS) (i.e. ACh-induced tone + EFS-induced force) increased over time and reached a plateau after about 50 min. However, the EFS-induced response alone was lower than the EFS-induced response in the absence of ACh-induced tone [30]. In the present model, the increase in basal tone was not associated with a reduction in the maximum response to exogenous ACh or an increase after 60 min of stretching. The main difference between the two models is that in the ovine tracheas, ACh is involved both in the increase in basal tone and the response to EFS; in contrast, the loading-induced increase in basal tone in our model was unlikely to have involved endogenous release of ACh [8], and the maximum response to ACh was obtained by cumulative addition of exogenous ACh. However, our results could be explained (at least in part) by the gradual transfer of the generated force to the cytoskeleton's passive structure through cross-bridges. This would increase the ASM's force during the 30-sec recovery phase. Moreover, the results of experiments on isolated airway strips and on ASM cells have suggested that if the imposed force fluctuations become too small, the ASM will stiffen due to decreased cross-bridge cycling and/or low cytoskeletal fluidization [31]. In contrast, when exposed to force fluctuations, the ASM's length corresponds to a dynamically equilibrated state that depends on the force's oscillation amplitude and frequency. This mechanism (derived from alterations in the dynamic features of breathing) is unique and is distinct from a change in the mean static load only. Moreover, computer modeling has shown that the application of length oscillations around the ASM's isometric length decreases the number of attached actin-myosin cross-bridges [32].

Our study had several strengths and limitations. One of the strengths relates to the fact that we used whole human bronchus preparations, thus providing a physiologically relevant *in vitro* environment in which airway responsiveness can be directly measured. Another strength relates to the use of a tension that corresponded to the intrinsic properties of each bronchus segment. In other words, loading human bronchi with excessive force would generate different results, and is more likely to stimulate intracellular signaling pathways via integrin activation and mechanotransduction [8,33,34]. Lastly, we used human bronchi, rather than bronchi from laboratory animals. This is important because there are clear interspecies differences in airway basal tone modulation and the control of airway responsiveness [18,35]. However, bronchi from patients undergoing thoracic surgery may have been stretched during the surgical procedure, which would limit the extrapolation of our results. Moreover, the tested bronchial rings were no longer connected to the extracellular matrix or the lung parenchyma, which might have counteracted (at least in part) the rise in basal tone and the effects of local mediators triggered by pulmonary stretch. The interaction between the steel hooks and the bronchial ring may cause local damage in the tissue. Nevertheless, the surface area of contact is negligible compared to the total surface area of the bronchial inner wall. Our system is largely used in the field of pharmacometrics and allows to fully measure the radial forces imposed on bronchus or vessel. Morover, the absence of decrease in responsiveness after cyclic loading in the present study suggests the absence of damage in the ASM.

## Conclusions

We developed a novel experimental device for investigating the effects of cyclic stretching on bronchial tone and responsiveness. Low-frequency, cyclic loading (imposing a maximum tension adjusted to the submaximum contractile response to exogenous ACh of each individual bronchial segment) is associated with an increase in basal tone and ACh responsiveness. The present integrated model (based on intact human bronchial rings) is likely to be a useful tool for future investigations of the bronchial response to repetitive stress during mechanical ventilation.
